# Resting‐State EEG Parameters Reveal Associations with AD Plasma Biomarkers in Cognitive Impairment

**DOI:** 10.1002/alz70856_102943

**Published:** 2025-12-26

**Authors:** Giordano Cecchetti, Giulia Rugarli, Luca Zanchi, Jacopo Lanzone, Silvia Basaia, Marco Cursi, Federico Coraglia, Edoardo G. Spinelli, Francesca Caso, Davide G. Curti, Giovanna F Fanelli, Anna Bellini, Giuseppe Magnani, Federica Agosta, Massimo Filippi

**Affiliations:** ^1^ IRCCS San Raffaele Scientific Institute, Milan, MI, Italy; ^2^ Vita‐Salute San Raffaele University, Milan, Italy; ^3^ IRCCS San Raffaele Scientific Institute, Milan, Italy; ^4^ University Vita‐Salute San Raffaele, Milan, MI, Italy; ^5^ Vita‐Salute San Raffaele University, Milan, MI, Italy

## Abstract

**Background:**

Alzheimer's disease (AD) is a neurodegenerative disorder characterized by cognitive decline and cortical dysfunction. Resting‐state EEG provides insights into AD‐related neurophysiological changes. This study investigates the associations between 32‐channel resting‐state EEG and AD plasma biomarkers in patients from a Memory Clinic population.

**Method:**

This cross‐sectional study consecutively included 193 patients with cognitive disturbances due to heterogeneous conditions from the tertiary Memory Clinic at San Raffaele Hospital, Milan, Italy. Resting‐state 32‐channel EEG and Lumipulse plasma biomarkers (pTau217, pTau181, NfL, Ab42/40 ratio) were collected. Patients were stratified into three groups based on dual pTau217 cutoffs, calculated on a sample of 184 individuals to achieve 97% sensitivity and specificity for identifying pathological CSF pTau181/Aβ42 ratios. Linear regression models, adjusted for age, sex, and disease duration, assessed associations between plasma biomarkers and EEG parameters, including alpha amplitude, alpha frequency, and relative power in delta, theta, alpha, beta, and gamma bands. ANCOVA compared EEG parameters across the three pTau217‐defined groups.

**Result:**

In the full sample, pTau217, pTau181, and the pTau217/Ab42 ratio were positively associated with theta power (+0.34 to +0.45) and delta power (+0.24), and negatively with alpha amplitude, alpha power (‐0.20 to ‐0.32), and beta power (‐0.20). In group 1 (low pTau217), the pTau217/Abeta42 ratio was negatively associated with beta, gamma, and alpha power (‐0.27 to ‐0.32). In group 2 (intermediate pTau217), NfL was positively associated with theta power (+0.47) and negatively with beta power (‐0.35). In group 3 (high pTau217), pTau217, pTau181, and the pTau217/Abeta42 ratio were positively associated with theta (+0.39) and delta power (+0.30), and negatively with alpha amplitude and alpha power. ANCOVA showed reduced alpha amplitude and alpha power together with higher theta in groups 2 and 3 versus group 1.

**Conclusion:**

This study demonstrates that resting‐state EEG parameters, particularly theta and alpha power, are closely linked to AD plasma biomarkers. Stratification based on pTau217 revealed distinct EEG patterns across biomarker‐defined groups, emphasizing EEG's utility as a non‐invasive tool for capturing disease‐specific neurophysiological alterations. These findings underline the potential of EEG as an accessible, non‐invasive, and scalable method to support the diagnosis and monitoring of AD.

**Funding**: Fujirebio Italia

